# 2323 Large patient volume is associated with adverse patient outcomes among those requiring maintenance renal replacement therapy

**DOI:** 10.1017/cts.2018.152

**Published:** 2018-11-21

**Authors:** Scott Reule, Robert Foley, Areef Ishani, Mark Rosenberg

**Affiliations:** 1 CTSI, University of Minnesota, Minneapolis, MN, USA; 2 Department of Medicine, University of Minnesota, Minneapolis, MN, USA; 3 Department of Medicine, Veterans Affairs Health Care System, Minneapolis, MN, USA

## Abstract

OBJECTIVES/SPECIFIC AIMS: We set out to describe important associations and outcomes among those requiring maintenance renal replacement therapy with the patient volume per provider. METHODS/STUDY POPULATION: Through the combination of several large administrative datasets, including the United States Renal Data System (n=237,485), the American Medical Association Master file (n=6249), and Medicare data limited to 2012, we compared characteristics of patients, by quintile of patient/provider volume. χ^2^ and logistic regression, adjusted for various patient and provider factors for categorical and continuous variables, was used for baseline comparisons, respectively. Cox regression, adjusted for patient, provider, and socioeconomic variables, was used to calculate risks for important clinical outcomes such as kidney transplant listing, transplant receipt, and all-cause mortality. RESULTS/ANTICIPATED RESULTS: There is a threshold patient volume at which important clinical outcomes, including kidney transplantation and all-cause mortality, may be influenced. Higher patient volume is associated with adverse patient outcome. Those receiving care from providers with the highest patient volumes are less likely to receive kidney transplantation, live in a more rural area, and be non-White. DISCUSSION/SIGNIFICANCE OF IMPACT: There is a need to identify novel and potentially modifiable factors associated with patient outcome among those with end-stage kidney disease on maintenance renal replacement therapy. Provider level variables, such as patient volume, is one such variable. As nephrologists are often tasked with the care of variable numbers of patients on dialysis, a better understanding of this association is an unmet need.

